# Reliability of the Mouse Grimace Scale in C57BL/6JRj Mice

**DOI:** 10.3390/ani10091648

**Published:** 2020-09-14

**Authors:** Katharina Hohlbaum, Giuliano Mario Corte, Melanie Humpenöder, Roswitha Merle, Christa Thöne-Reineke

**Affiliations:** 1 Institute of Animal Welfare, Animal Behavior and Laboratory Animal Science, Department of Veterinary Medicine, Freie Universität Berlin, 14163 Berlin, Germany; Melanie.Humpenoeder@fu-berlin.de (M.H.); Thoene-Reineke.Christa@fu-berlin.de (C.T.-R.); 2 Institute of Veterinary Anatomy, Department of Veterinary Medicine, Freie Universität Berlin, 14195 Berlin, Germany; giuliano.corte@fu-berlin.de; 3 Institute for Veterinary Epidemiology and Biostatistics, Department of Veterinary Medicine, Freie Universität Berlin, 14163 Berlin, Germany; Roswitha.Merle@fu-berlin.de

**Keywords:** laboratory mice, animal welfare indicators, Mouse Grimace Scale, intraclass correlations coefficient, Fleiss’ kappa, Cohen’s kappa

## Abstract

**Simple Summary:**

The protection and welfare of laboratory animals is ethically and legally required. Any possible impairment of well-being caused by husbandry or experiment must be reduced to a minimum. A prerequisite for ameliorating animal welfare is that we recognize the current well-being state of the animals by using measuring tools, for instance, the grimace scale. Facial expressions of mice, the most frequently used laboratory animals, can be analyzed using the Mouse Grimace Scale, which consists of five action units, i.e., orbital tightening, nose and cheek bulge, ear position, and whisker change. Since it is important that the animal welfare measuring tools are reliable and objective, we investigated the reliability of the Mouse Grimace Scale. Our results indicated good agreement between all observers applying the Mouse Grimace Scale. However, when investigating the action units individually, the best agreement was achieved for orbital tightening and the poorest agreement for nose as well as cheek bulge, depending on the observers’ experience levels. Against this background, we critically discuss factors that potentially influence the reliability of the Mouse Grimace Scale to improve its application.

**Abstract:**

To maintain and foster the welfare of laboratory mice, tools that reliably measure the current state of the animals are applied in clinical assessment. One of these is the Mouse Grimace Scale (MGS), a coding system for facial expression analysis. Since there are concerns about the objectivity of the MGS, we further investigated its reliability. Four observers (two experienced and two inexperienced in use of the MGS) scored 188 images of 33 female and 31 male C57BL/6JRj mice. Images were generated prior to, 150 min, and two days after ketamine/xylazine anesthesia. The intraclass correlations coefficient (ICC = 0.851) indicated good agreement on total MGS scores between all observers when all three time points were included in the analysis. However, interrater reliability was higher in the early post-anesthetic period (ICC = 0.799) than at baseline (ICC = 0.556) and on day 2 after anesthesia (ICC = 0.329). The best agreement was achieved for orbital tightening, and the poorest agreement for nose and cheek bulge, depending on the observers’ experience levels. In general, experienced observers produced scores of higher consistency when compared to inexperienced. Against this background, we critically discuss factors that potentially influence the reliability of MGS scoring.

## 1. Introduction

The protection and welfare of laboratory animals is ethically and legally required [1]. Any possible pain, suffering, distress, and lasting harm caused by husbandry or experiment must be reduced to a minimum [1], which also contributes to the quality of research findings [2]. A prerequisite for ameliorating animal welfare is that we recognize the current well-being state of an individual. When impaired well-being is identified, the animal can be treated accordingly.

The assessment of well-being is a serious challenge, especially in mice, the most commonly used laboratory animals in biomedical research. Mice are prey animals and therefore may often hide signs of pain, injury, and weakness when humans are present [3]. This underlines that a major current animal welfare issue is to develop and further refine tools that reliably measure the well-being of an animal. Humans have a tendency to focus on the face of an animal when assessing its current well-being [4]. Indeed, changes in facial expressions can indicate pain as shown for mice [5] and other animals such as rats [6], rabbits [7], cats [8,9], ferrets [10], sheep [11], piglets [12], cows [13], and horses [14]. However, grimacing can also be induced by other factors. For instance, the facial expression of mice can be affected by vibrissae contact, social proximity, cat odor exposure, rat exposure, aggression, or subordination in the resident–intruder test, and anesthesia [15,16,17]. To facilitate facial expression analysis in mice, the Mouse Grimace Scale (MGS) was developed [5] and has become a valuable tool in laboratory animal science in recent years; it is used in biomedical research as well as in cage-side clinical assessment [18,19]. The MGS includes five so-called facial action units (FAU), i.e., orbital tightening, nose bulge, cheek bulge, ear position, and whisker change [5]. The presence and intensity of these features can provide an insight into the affective state of a mouse.

Experts in laboratory animal welfare consider the MGS as an important welfare indicator [20]. However, concerns about the reliability of the MGS are frequently raised because, at this point, it is usually applied by human observers and, therefore, MGS scores are assumed to be subjective to a certain extent.

We had similar experiences in a previous study where four persons, who had a professional background in animal science but different experiences in the use of the MGS, were recruited to score images of C57BL/6JRj mice generated before and after ketamine/xylazine anesthesia [17]. After the MGS scoring in Hohlbaum et al. (2018) [17], the four observers reported that they found some of the FAU units difficult to score (personal communication).

Therefore, as a follow-up to our previous study [17], we conducted an interrater reliability analysis to determine whether the MGS data reflected the scorers’ subjective impression. We also investigated interrater effects on the individual facial features and critically discussed factors that may influence the reliability of the MGS scoring.

## 2. Methods

### 2.1. Animals and Handling Methods

A total number of 64 adult C57BL/6JRj mice (33 females, 31 males; 10–13 weeks of age) were obtained from Janvier Labs (Saint-Berthevin Cedex, France) [17]. Sample size was calculated for our previous study on the impact of repeated ketamine/xylazine anesthesia on the well-being of mice and can be found in Hohlbaum et al. [17]. Animals were kept in a conventional (non-SPF) facility and were free of all viral, bacterial, and parasitic pathogens listed in the FELASA (Federation for Laboratory Animal Science Associations) recommendations [21]. Females were kept in groups of three to five animals in Makrolon type IV cages (55 × 33 × 20 cm); males had to be kept individually in Makrolon type III cages (42 × 26 × 15 cm) because of aggressive behavior [17]. The cages were filled with fine wooden bedding material (LIGNOCEL^®^ 3-4 S, J. Rettenmaier & Söhne GmbH + Co. KG, Rosenberg, Germany), nestlets (Ancare, UK agents, Lillico, United Kingdom), a red plastic house (length: 100 mm, width: 90 mm, height: 55 mm; ZOONLAB GmbH, Castrop-Rauxel, Germany), and metal tunnels (length: 125 mm, diameter: 50 mm; one tunnel in Makrolon type III cages, two tunnels in Makrolon type IV cages). The animals were maintained under standard conditions (room temperature: 22 ± 2 °C; relative humidity: 55 ± 10%) on a light:dark cycle of 12:12 h of artificial light with a 5 min twilight transition phase (lights on from 6:00 a.m. to 6:00 p.m.) [17]. Pelleted mouse diet was fed ad libitum (Ssniff rat/mouse maintenance, Spezialdiäten GmbH, Soest, Germany) [17]. The mice had free access to tap water [17]. Animals were handled using a combination of the tunnel and cup techniques [22]. After the experiment was completed, female animals were rehomed; male mice were either used for educational purposes or castrated and resocialized in stable groups to be rehomed.

### 2.2. Anesthesia

Detailed description of anesthetic and monitoring procedures is available in Hohlbaum et al. [17]. In brief, a dosage of 80 mg/kg ketamine and 16 mg/kg xylazine (Ketavet^®^ 100 mg/mL, Zoetis Deutschland GmbH, Berlin, Germany; Rompun^®^ 2%, Bayer Vital GmbH, Leverkusen, Germany) was administered intraperitoneally at a volume of 10 μL/g body weight using 27 ¾ gauge needles [17]. After the loss of the righting reflex, mice were placed in abdominal position on a heating pad. The temperature of the heating pad could be adjusted from 30–42 °C. Artificial tears (Artelac^®^ Splash MDO^®^, Bausch & Lomb GmbH, Berlin, Germany) were administered to the eyes [17]. Reflexes and vital parameters were carefully monitored during anesthesia, as reported in Hohlbaum et al. [17].

The mice were either anesthetized once (13 females, 13 males) or six times (i.e., every three to four days; 13 females, 12 males). In the latter case, images generated after their last anesthesia were examined. Control animals (7 females, 6 males) did not receive anesthesia. Since the effects of the different anesthesia regimes on the well-being of the animals had already been investigated in Hohlbaum et al. [17] and are outside the focus of this article, we do not differentiate between the treatment groups in the present article.

### 2.3. Mouse Photography and Grimace Scale Scoring

MGS scores were obtained from photographs (n = 188; four female mice were excluded on day 2 due to technical malfunction of the camera) generated two days prior to anesthesia (baseline), at 150 min, and two days after anesthesia, as previously described [17]. In brief, photographs were generated in a custom-made box (22 × 29 × 39 cm, three white walls, one transparent wall, 0.5 cm bedding material including soiled bedding) using a high definition camera (Canon EOS 350D, Canon Inc., Tokyo, Japan) [23].

The observers were blinded to the time point of image generation. Among them were three veterinarians and one animal technician. Due to their professional backgrounds, all of them were highly familiar with animal behavior. Scorers 1 and 2 had no previous experience in using the MGS scale, scorers 3 and 4 had already gathered experience in MGS scoring for approximately eleven months. They received electronic files containing the mouse images [23] and scored them according to the MGS at a time that was convenient to them and at their own pace (i.e., the scorers did not simultaneously evaluate the images). In brief, the five FAU (i.e., orbital tightening, ear position, whisker change, nose bulge, and cheek bulge,) were scored on a 3-point scale from 0 to 2 (0 = not present, 1 = moderately present, 2 = obviously present) [5]. An additional score “not assessable” was introduced for those cases in which a facial action unit could not be scored. Each observer scored 188 images that were then further analyzed as follows: The scores of the five facial action units were added up (overall MGS scores ranging from 0 to 10) and averaged (mean MGS scores), whereby facial action units scored “not assessable” were excluded. To calculate the mean MGS difference score, baseline MGS scores were subtracted from MGS scores obtained after anesthesia.

It should be noted that the score “not assessable” was included for scores of the individual FAUs, but excluded for overall MGS scores, mean MGS scores, as well as mean MGS difference scores.

### 2.4. Statistical Analysis

To assess the interrater reliability between the four scorers, weighted Fleiss’ kappa (κ) statistics or intraclass correlation coefficients (ICC) were used, depending on whether the data were measured on an ordinal or continuous scale [24]. The single facial action units were scored on a 3-point scale, resulting in ordinal data. Hence, the weighted Fleiss’ kappa was the most appropriate reliability parameter. However, since other working groups reported the ICC instead of the Fleiss’ kappa, we also analyzed the ICC (model: two-way random model with observer as random factor, type: absolute agreement) for each facial action unit, in order to be able to compare the data. Overall MGS scores, mean MGS scores, and mean MGS differences scores were considered to be continuous and were analyzed using the ICC (model: two-way random model with observer as random factor, type: absolute agreement). Interrater reliability for the two inexperienced and the two experienced scorers was determined using Cohen’s kappa.

ICC values were evaluated according to Koo and Li [25]:

- <0.50 poor interrater reliability
- 0.50–0.75 moderate interrater reliability
- 0.75–0.90 good interrater reliability
- >0.90 excellent interrater reliability

Fleiss’ kappa (κ) and Cohen’s kappa was interpreted using guidelines stated by Landis and Koch [26]:

- <0 poor interrater reliability
- 0.00–0.20 slight interrater reliability
- 0.21–0.40 fair interrater reliability
- 0.41–0.60 moderate interrater reliability
- 0.61–0.80 substantial interrater reliability
- 0.81–1 almost perfect interrater reliability

Statistical analysis was performed with IBM SPSS Version 25 and Version 26 (IBM Corporation, Armonk, NY, USA). An extension of IBM SPSS was used for Fleiss’ kappa statistics [27]. Differences between facial action units were analyzed using Friedman’s two-way analysis of variance by ranks with a significance level of 0.05.

### 2.5. Ethical Approval and Informed Consent

The study was performed according to the guidelines of the German Animal Welfare Act and the Directive 2010/63/EU for the protection of animals used for scientific purposes. Maintenance of mice and all animal experimentation were approved by the Berlin State Authority (“Landesamt für Gesundheit und Soziales”, permit number: G0053/15). The purpose of the study was to investigate the impact of repeated ketamine/xylazine anesthesia on the well-being of C57BL/6JRj mice [17]. In the present work, we investigated and discussed the MGS scores obtained in this study in more detail.

## 3. Results

**Figure 1:**
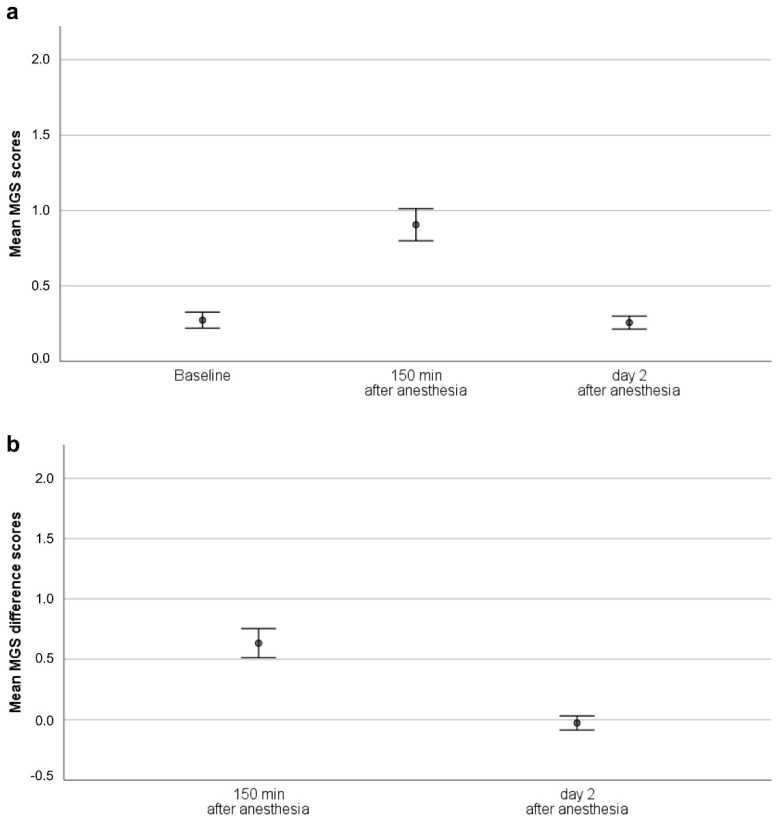
MGS scores (mean, 95% confidence interval). (**a**) Mean MGS scores, (**b**) Mean MGS difference scores. Please note that data were derived from animals that received either no, one, or six anesthesia. Since the present study focuses on MGS reliability, treatment groups are not differentiated. The effects of the anesthesia regimes on the well-being of mice in the different treatment groups was investigated in a previous study by Hohlbaum et al. [17]. Abbreviation: Mouse Grimace Scale (MGS).

**Figure 2:**
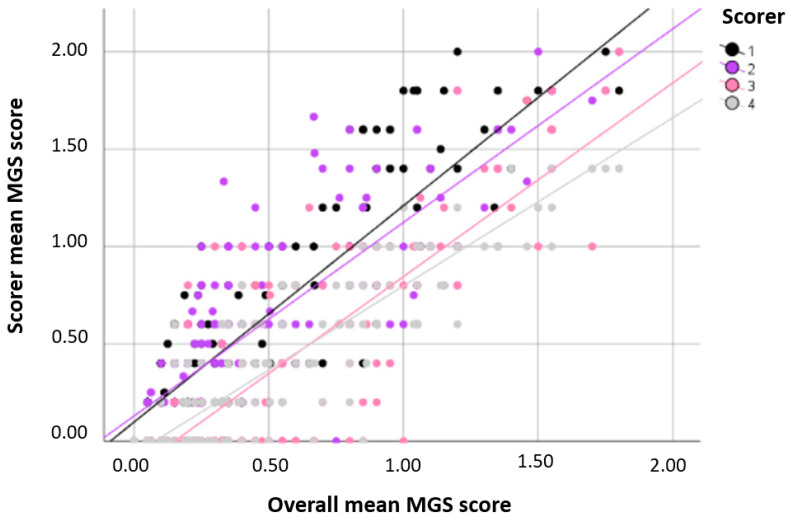
Reliability analysis of the mean MGS scores. The mean MGS scores of each of the four scorers were compared to the mean MGS scores of all scorers. Three scores for each mouse were included in the analysis (i.e., one score each was obtained from images generated at baseline, 150 min, and two days after anesthesia). Abbreviation: Mouse Grimace Scale (MGS).

For overall MGS scores (i.e., total MGS scores, mean MGS scores, and mean MGS difference score; Figure 1), the intraclass correlation coefficient (ICC) revealed good agreement (Table 1) when scores of each mouse obtained at baseline, 150 min, and two days after anesthesia were included in the analysis. When the time points were analyzed separately, interrater reliability deteriorated at baseline (moderate) and on day 2 after anesthesia (poor), but remained good at 150 min after anesthesia (Table 1). While experienced scorers reached good agreement in overall MGS scores over all time points, those from inexperienced scorers indicated moderate to good agreement (Table 1). The ICC values over all time points did not improve by excluding the scores of any scorer. Figure 2 illustrates the reliability of the mean MGS scores. Interestingly, the MGS scores obtained from scorers 1 and 2 appeared to be higher than those generated by scorers 3 and 4 (Figure 2).

Fleiss’ kappa indicated substantial agreement between all four observers for orbital tightening, and fair agreement for ear position as well as whisker change, while slight agreement was reached for nose and cheek bulge, when scores of all time points were included in the analysis (Table 2). In some cases, the interrater reliability of the individual FAU scores differed between the three time points at which the images were acquired. Fleiss’ kappa revealed a higher consistency in scores of orbital tightening and whisker change at 150 min after anesthesia (orbital tightening: moderate; whisker change: fair) than at baseline or on day 2 (orbital tightening: fair; whisker change: slight) (Table 2). The consistency in nose and cheek bulge scores were slight at baseline and 150 min after anesthesia but poor on day 2. Independent of the time point, ear position scores showed slight agreement.

Interrater reliability was separately analyzed for inexperienced and experienced observers using Cohen’s kappa. Both inexperienced and experienced scorers achieved substantial agreement for orbital tightening. Moderate agreement between experienced observers for ear position as well as whisker change and fair agreement for nose and cheek bulge were achieved, whereas agreement between inexperienced observers was only fair for ear position as well as whisker change and slight for cheek and nose bulge.

## 4. Discussion

Due to ethical, scientific, and legal issues [1,2], it is crucial to develop scientifically sound tools to assess the well-being of mice so that those with disturbed well-being can be identified and treated accordingly. This implies that the tools can be easily applied in practice and reliably measure welfare. Since there are concerns about the objectivity of the MGS, we took a closer look at its reliability when scoring images of C57BL/6JRj mice generated prior to and after ketamine/xylazine anesthesia. We found good agreement between observers for overall MGS scores (i.e., including orbital tightening, nose bulge, cheek bulge, ear position, and whisker change), though the interrater reliability depended on the affective state of the mice, the observers’ experience levels, and the scored facial features. The best agreement was achieved for orbital tightening, the poorest agreement for nose and cheek bulge.

The MGS was originally developed to identify acute pain from the facial expressions of mice [5], however, grimacing can also be induced by other stress factors [15,16,17]. In the present study, we assume that changes in facial expressions were primarily due to pharmacological effects of the injection anesthetics [17]. The fact that the mice did not experience acute pain is not relevant for this study, because it should not influence the interrater reliability. For instance, if a certain FAU does not change after anesthesia, the scorers should be able to recognize the lack of facial expression changes and assign appropriate scores. Nevertheless, against the background that anesthesia may cause lower MGS scores than acute pain, i.e., less obvious facial expression changes, it is conceivable that interrater reliability may be higher for faces of mice experiencing acute pain.

Our interrater reliability of mean MGS scores in C57BL/6JRj mice (ICC = 0.845 over all time points) was similar to that found by the MGS inventors (ICC = 0.90) in CD-1 mice [5] and other working groups in CD-1 mice (ICC = 0.8405) [28], BALB/c mice (ICC ranging from 0.75 to 0.84) [29], or BALB/cAnNCrl mice (ICC ranging from 0.7 to 0.84) [30]. In cats, the ICC between four persons applying the Feline Grimace Scale was 0.84 [31].

In the present study, the ICC values comparing overall MGS scores acquired from inexperienced or experienced observers did not improve by excluding the scores of any scorer, suggesting that the observers evaluated the images consistently.

Interestingly, images generated at 150 min after anesthesia were scored with a higher consistency when compared to baseline images or those acquired on day 2 after anesthesia. This phenomenon was particularly noticeable regarding orbital tightening as well as whisker change and indicated that facial expressions of C57BL/6JRj mice with undisturbed well-being, i.e., at baseline or on day 2, seemed more difficult to score. Vice versa, changes in facial expressions caused by the experimental treatment were easier recognizable for the observers, as expected above.

To investigate the reported difficulty of scoring certain facial features in the images of C57BL/6JRj mice, we analyzed the interrater reliability separately for the four observers and each FAU using Fleiss’ kappa and found widely divergent results. The highest agreement was observed for orbital tightening, followed by ear position and whisker change, whereas cheek and nose bulge scorings were less homogeneous among the four observers. To be able to compare our findings to Costa et al. [28], who described the ICC instead of Fleiss’ kappa for all FAU, we also calculated the ICC. It was not possible to measure the Fleiss’ kappa for the data given in Costa et al. because in their study not all observers scored all images on the MGS. While the interrater reliability was similar for orbital tightening (ICC = 0.858) and only slightly different for ear position (ICC = 0.756), Costa et al. measured much higher values for nose (ICC = 0.557) and cheek bulge (ICC = 0.644).

This discrepancy may be explained by different factors that can either be observer-dependent (experience, training), method-dependent (image quality, mutual interference of FAU, level of difficulty to score a FAU), animal-dependent (recognizability of FAU due to fur color), or treatment-dependent (height of MGS scores).

Langford et al. previously demonstrated that the observers’ experience levels in the use of the MGS can have critical influence on the accuracy of the MGS [5]. An accuracy of 97% and 81% were determined for experienced and inexperienced scorers, respectively [5]. The accuracy was shown to deteriorate when low instead of high resolution images were presented to the observers [5], which emphasized the importance of image quality for the MGS application. In our study, two inexperienced observers and two persons with 11-month experience in MGS scoring were recruited to score images with a very high resolution (3.456 × 2.304 pixels). A separate analysis of the interrater reliability of experienced and inexperienced scorers revealed that the first achieved higher agreements for overall MGS scores and for almost all FAU, except for whisker change. Experienced persons from our study reached similar ICC values for orbital tightening, nose bulge, cheek bulge, and ear position when compared to Costa et al. [28]. From this, we hypothesized that consistency in MGS scoring may increase with the observers’ experience and, therefore, the level of experience can strongly affect the interrater correlation. However, Roughan and Sevenoaks found equal or even greater consistency in the scores generated for BALB/c mice by inexperienced observers when compared to experienced observers [30]. Novices may have performed worse in our study than in the latter work because the MGS manual and MGS poster provided by the NC3Rs (National Centre of Replacement, Refinement, and Reduction, United Kingdom of Great Britain) is limited to white-furred mice [5,32]. Therefore, the inexperienced scorers in our study were confronted with the difficulty of transferring facial expressions of white-furred to black-furred mice.

Another interesting observation to note is that the inexperienced scorers generally assigned higher scores to the mouse images. This may be explained by initial uncertainties in MGS scorings, which may tempt them to orient towards the intensity of easily accessible facial features when applying the MGS. Thus, the score of a FAU can easily be biased by the intensity of other FAU. This is not surprising, as humans have a tendency to focus on particular facial features in order to assess the affective state of another person, whereby the features of interest depend on the respective state [33]. A template covering all the other facial features may solve this problem but would increase the labor costs for MGS scoring.

Furthermore, we deem the training process important and postulate that the method used to introduce new scorers to the MGS may significantly influence interrater reliability. In our study, the two experienced scorers received one-to-one training; the two inexperienced scorers were trained together and had the opportunity to discuss their scores with the trainer as well as the other trainee. Against this background, we assumed the group training would produce a higher agreement between the scorers, which was not true in our study. The interrater reliability may be improved if trainees perform multiple training sessions, and if scores from the first round are reviewed and discussed with an expert in the second round [34]. Apart from this, Zhang et al. demonstrated a beneficial effect for the group discussion about the grimace scale scores that were assigned to the images [34]. As the grimace scale training varies between laboratories, ranging from reviewing the grimace scale manual to a single or multiple training sessions of varying length and intensity (e.g., one-to-one or group training including discussions), the effectiveness of the respective training and the actual proficiency should be tested [5,34,35,36,37].

Since agreement between inexperienced observers was highest for orbital tightening and lowest for nose as well as cheek bulge, we suggested that the former was the easiest and the latter were the hardest to evaluate. A reason for this may be the degree of detail recognition, which is higher for those facial features colored in contrast to the fur. In C57BL/6JRj mice, the ears are lighter- and eyes are darker-colored than the fur and the whiskers contrast well against the whitish background. The background of the images acquired in our study was white, because we utilized a custom-made photography cube with one clear and three white walls. In contrast to ears, eyes, and whiskers, the cheeks and nose bridge have the same color as the faces of the mice. In other species such as cats and horses, it has also been reported that the identification of some FAU is difficult in black-furred animals [8,14,31].

Evaluations of the interrater reliability in Rat Grimace Scale scoring revealed that orbital tightening had the highest and whisker change had the lowest ICC value [34]. Probably due to the difficulty in clearly recognizing and evaluating certain FAU, some working groups excluded nose and cheek bulge [19] or whisker change [38,39] from facial expression analysis in mice. Similar observations were made in cats: the highest ICC value was found for orbital tightening and the lowest for muzzle tension as well as whisker change [31]. Considering these challenges of scoring certain FAU, grimace scales that were developed more recently often comprise less parameters than earlier grimace scales.

Another strategy to consider the differences in interrater reliability would be to add different weights to the five FAU, depending on the degree of agreement.

The questions that have arisen cannot be investigated based on our data, as the number of scorers is too low and we did not follow up their progress in MGS scoring. To further improve the reliability of MGS, the issues raised need to be addressed. A further approach to generate more objective and reliable data is to standardize the experimental set-up for MGS scoring, including image/video generation, training, as well as scoring sessions, and to automatize the facial expression analysis of mice [40,41,42].

## 5. Conclusions

Coming back to our initial questions: Did the MGS data represent the observers’ subjective impressions that some of the FAU units were difficult to score? In summary, we found that nose and cheek bulge appeared to be FAU with potential difficulties, especially for the inexperienced observers in our study. Moreover, facial expressions that were affected by the anesthetic treatment were scored more consistently than those without pharmacological influence. However, although our analysis is based on a large image dataset, we could only recruit four scorers, which is why the conclusion drawn in this article is limited and should be verified by further studies.

## Figures and Tables

**Table 1:** Interrater reliability of overall MGS scores.

	Total MGS Scores ^1^	Mean MGS Scores ^1^	Mean MGS Difference Scores ^2^
**Interrater Reliability between All Scorers**			
ICC over all time points ^3^	0.851	0.845	0.855
ICC at baseline	0.556	0.522	-
ICC at 150 min after anesthesia	0.799	0.802	0.813
ICC on day 2 after anesthesia	0.329	0.317	0.433
**Interrater Reliability between Inexperienced Scorers**			
ICC over all time points ^3^	0.749	0.757	0.736
**Interrater Reliability between Experienced Scorers**			
ICC over all time points ^3^	0.824	0.821	0.811
**Interrater Reliability between Three Scorers, Excluding One Person at a Time**			
ICC over all time points ^3^, excluding scorer #1 (inexperienced)	0.812	0.797	0.791
ICC over all time points ^3^, excluding scorer #2 (inexperienced)	0.826	0.822	0.855
ICC over all time points ^3^, excluding scorer #3 (experienced)	0.814	0.808	0.816
ICC over all time points ^3^, excluding scorer #4 (experienced)	0.791	0.786	0.799

^1^ Scores obtained at baseline, 150 min, and two days after anesthesia. ^2^ Scores obtained at 150 min, and two days after anesthesia. Baseline scores were subtracted from the score generated after anesthesia. ^3^ Three scores of each mouse were included in the analysis (i.e., one score each was obtained from images generated at baseline, 150 min, and two days after anesthesia). Abbreviations: Intraclass correlation (ICC), Mouse Grimace Scale (MGS).

**Table 2:** Interrater reliability of each facial action unit.

	Orbital Tightening	Nose Bulge	Cheek Bulge	Ear Position	Whisker Change
**Interrater Reliability between All Scorers**					
Fleiss’ kappa over all time points ^1^	0.664	0.093	0.125	0.285	0.279
Intraclass correlation over all time points ^1,2^	0.876	0.156	0.146	0.542	0.519
Fleiss’ kappa at baseline	0.245	0.104	0.032	0.164	0.066
Intraclass correlation at baseline ^2^	0.416	0.117	0.041	0.173	0.174
Fleiss’ kappa at 150 min after anesthesia	0.531	0.052	0.083	0.197	0.220
Intraclass correlation at 150 min after anesthesia ^2^	0.900	0.243	0.115	0.674	0.721
Fleiss’ kappa on day 2 after anesthesia	0.357	−0.089	−0.031	0.074	0.096
Intraclass correlation on day 2 after anesthesia ^2^	0.695	0.098	0.102	0.282	0.450
**Interrater Reliability between Inexperienced Scorers**					
Cohen’s kappa over all time points ^1^	0.708	0.197	0.013	0.280	0.244
**Interrater Reliability between Experienced Scorers**					
Cohen’s kappa over all time points ^1^	0.629	0.355	0.372	0.484	0.428

The score “not assessable” was included in the analysis. ^1^ Three scores of each mouse were included in the analysis (i.e., one score each was obtained from images generated at baseline, 150 min, and two days after anesthesia). ^2^ To be able to compare our data with other studies, we calculated the ICC in addition to Fleiss’ kappa. Abbreviation: Intraclass correlation (ICC).
